# *N*-Isopropyl-3-methyl-2-nitro­benzamide

**DOI:** 10.1107/S1600536809031997

**Published:** 2009-08-29

**Authors:** Yu Chen, Li-hua Guo, Wei Song, Jing Zhang, Dan-bi Tian

**Affiliations:** a Department of Applied Chemistry, College of Science, Nanjing University of Technology, Nanjing 210009, People’s Republic of China

## Abstract

In the title compound, C_11_H_14_N_2_O_3_, the bond lengths and angles are within normal ranges. Weak inter­molecular N—H⋯O inter­actions link the mol­ecules into chains along the *a* axis. A non-classical intra­molecular C—H⋯O inter­action (nitro O atom and a H atom of the nearest methyl group) is found, forming a six-membered ring with a twisted conformation. This six-membered ring has a twisted conformation.

## Related literature

For bond–length data, see: Allen *et al.* (1987 ▶). For general background, see: Lahm *et al.* (2005 ▶).
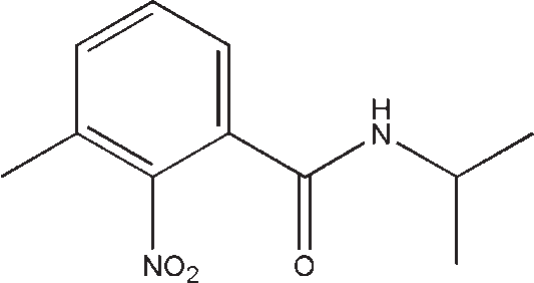


## Experimental

### 

#### Crystal data

C_11_H_14_N_2_O_3_
*M* *_r_* = 222.24
Orthorhombic, 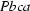

*a* = 9.4230 (19) Å
*b* = 13.250 (3) Å
*c* = 20.041 (4) Å
*V* = 2502.2 (9) Å^3^
*Z* = 8
Mo *K*α radiation
μ = 0.09 mm^−1^
*T* = 298 K
0.30 × 0.20 × 0.10 mm

#### Data collection

Enraf–Nonius CAD-4 diffractometer
Absorption correction: ψ scan (North *et al.*, 1968 ▶) *T*_min_ = 0.974, *T*_max_ = 0.991
2260 measured reflections
2260 independent reflections
1135 reflections with *I* > 2σ(*I*)
3 standard reflections every 200 reflections intensity decay: 1%

#### Refinement

*R*[*F* ^2^ > 2σ(*F* ^2^)] = 0.066
*wR*(*F* ^2^) = 0.174
*S* = 1.00
2260 reflections
146 parameters
H-atom parameters constrained
Δρ_max_ = 0.22 e Å^−3^
Δρ_min_ = −0.16 e Å^−3^

### 

Data collection: *CAD-4 EXPRESS* (Enraf–Nonius, 1994 ▶); cell refinement: *CAD-4 EXPRESS*; data reduction: *XCAD4* (Harms & Wocadlo, 1995 ▶); program(s) used to solve structure: *SHELXS97* (Sheldrick, 2008 ▶); program(s) used to refine structure: *SHELXL97* (Sheldrick, 2008 ▶); molecular graphics: *SHELXTL* (Sheldrick, 2008 ▶); software used to prepare material for publication: *SHELXTL*.

## Supplementary Material

Crystal structure: contains datablocks I, global. DOI: 10.1107/S1600536809031997/rk2153sup1.cif

Structure factors: contains datablocks I. DOI: 10.1107/S1600536809031997/rk2153Isup2.hkl

Additional supplementary materials:  crystallographic information; 3D view; checkCIF report

## Figures and Tables

**Table 1:** Hydrogen-bond geometry (Å, °)

*D*—H⋯*A*	*D*—H	H⋯*A*	*D*⋯*A*	*D*—H⋯*A*
N1—H1*A*⋯O1^i^	0.86	2.00	2.855 (3)	173
C11—H11*A*⋯O3	0.96	2.37	3.021 (5)	124

Symmetry code: (i) 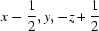.

**Fig. 1.**
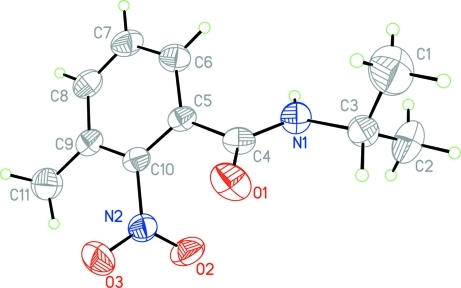
Asymmetric unit of the title molecular structure, with the atom–labeling scheme. Displacement ellipsoids are drawn at the 50% probability level. H atoms are presented as a small spheres of arbitrary radius.

**Fig. 2.**
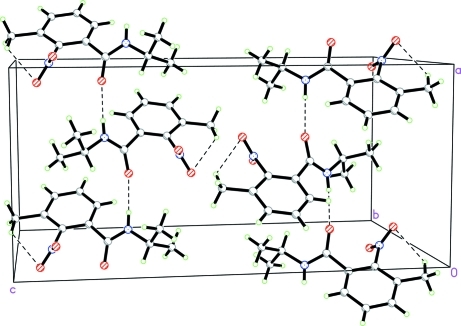
A packing diagram for (I).

## supplementary crystallographic information

### Comment

The title compound contains nitro– and acetylamino–groups, which can react with different groups to prepare various functional organic compounds as a fine organic intermediate (Lahm *et al.*, 2005). We herein report the crystal structure.

In the title molecule (I), (Fig. 1), the bond lengths and angles are within normal ranges (Allen *et al.*, 1987). Intramolecular C—H···O interaction (Table 1) results in the formation of a six–membered ring (O3/N2/C10/C9/C11/H11A), having twisted conformation.

In the crystal structure, weak intermolecular N—H···O interactions (Table 1) link the molecules into chains along the *a* axis (Fig. 2), in which they may be effective in the stabilization of the structure.

### Experimental

*N*–isopropyl–3–methyl–2–nitrobenzamide were dissolved in *DMF* (50 mL). The solution was then poured to ice water. The crystalline product was isolated by filtration, washed with water (600 ml), dried and gave the product 1.8 g. The single crystals were obtained by evaporating the acetone slowly at room temperature for about 14 d.

### Refinement

The H atoms were positioned geometrically, with N—H = 0.86 Å (for NH) and C—H = 0.93, 0.96 and 0.98 Å for aromatic, methyl and methine H atoms, respectively, and constrained to ride on their parent atoms, with *U*_iso_(H) = *xU*_eq_(C), where *x* = 1.5 for methyl H, and *x* = 1.2 for other H atoms.

### Crystal data

**Table:**

C_11_H_14_N_2_O_3_	*F*(000) = 944
*M**_r_* = 222.24	*D*_x_ = 1.180 Mg m^−^^3^
Orthorhombic, *P**b**c**a*	Mo *K*α radiation, λ = 0.71073 Å
Hall symbol: -P 2ac 2ab	Cell parameters from 25 reflections
*a* = 9.4230 (19) Å	θ = 10–13°
*b* = 13.250 (3) Å	µ = 0.09 mm^−^^1^
*c* = 20.041 (4) Å	*T* = 298 K
*V* = 2502.2 (9) Å^3^	Needle, colourless
*Z* = 8	0.30 × 0.20 × 0.10 mm

### Data collection

**Table:**

Enraf–Nonius CAD-4 diffractometer	1135 reflections with *I* > 2σ(*I*)
Radiation source: fine–focus sealed tube	*R*_int_ = 0.0000
graphite	θ_max_ = 25.3°, θ_min_ = 2.0°
ω/2θ scans	*h* = 0→11
Absorption correction: ψ scan (North *et al.*, 1968)	*k* = 0→15
*T*_min_ = 0.974, *T*_max_ = 0.991	*l* = 0→24
2260 measured reflections	3 standard reflections every 200 reflections
2260 independent reflections	intensity decay: 1%

### Refinement

**Table:**

Refinement on *F*^2^	Secondary atom site location: difference Fourier map
Least-squares matrix: full	Hydrogen site location: inferred from neighbouring sites
*R*[*F*^2^ > 2σ(*F*^2^)] = 0.066	H-atom parameters constrained
*wR*(*F*^2^) = 0.174	*w* = 1/[σ^2^(*F*_o_^2^) + (0.06*P*)^2^ + 0.9*P*] where *P* = (*F*_o_^2^ + 2*F*_c_^2^)/3
*S* = 1.00	(Δ/σ)_max_ < 0.001
2260 reflections	Δρ_max_ = 0.22 e Å^−^^3^
146 parameters	Δρ_min_ = −0.16 e Å^−^^3^
0 restraints	Extinction correction: *SHELXL97* (Sheldrick, 2008), Fc^*^=kFc[1+0.001xFc^2^λ^3^/sin(2θ)]^-1/4^
Primary atom site location: structure-invariant direct methods	Extinction coefficient: 0.0059 (11)

### Special details

**Table:**

Geometry. All s.u.'s (except the s.u. in the dihedral angle between two l.s. planes) are estimated using the full covariance matrix. The cell s.u.'s are taken into account individually in the estimation of s.u.'s in distances, angles and torsion angles; correlations between s.u.'s in cell parameters are only used when they are defined by crystal symmetry. An approximate (isotropic) treatment of cell s.u.'s is used for estimating s.u.'s involving l.s. planes.
Refinement. Refinement of *F*^2^ against ALL reflections. The weighted *R*–factor *wR* and goodness of fit *S* are based on *F*^2^, conventional *R*–factors *R* are based on *F*, with *F* set to zero for negative *F*^2^. The threshold expression of *F*^2^ > σ(*F*^2^) is used only for calculating *R*–factors(gt) *etc*. and is not relevant to the choice of reflections for refinement. *R*–factors based on *F*^2^ are statistically about twice as large as those based on *F*, and *R*–factors based on ALL data will be even larger.

### Fractional atomic coordinates and isotropic or equivalent isotropic displacement parameters (Å^2^)

**Table:**

	*x*	*y*	*z*	*U*_iso_*/*U*_eq_	
O1	0.1002 (2)	0.4262 (2)	0.21752 (13)	0.0801 (9)	
N1	−0.0987 (3)	0.4490 (2)	0.27836 (16)	0.0642 (9)	
H1A	−0.1881	0.4367	0.2809	0.077*	
C1	0.0066 (6)	0.4504 (4)	0.3892 (3)	0.1265 (19)	
H1B	0.0699	0.3974	0.3756	0.190*	
H1C	−0.0769	0.4215	0.4089	0.190*	
H1D	0.0533	0.4928	0.4212	0.190*	
O2	−0.0336 (3)	0.51283 (19)	0.09602 (15)	0.0890 (10)	
N2	0.0028 (3)	0.4269 (2)	0.08678 (16)	0.0635 (8)	
C2	−0.1321 (5)	0.5952 (3)	0.3487 (2)	0.1034 (16)	
H2A	−0.1543	0.6345	0.3099	0.155*	
H2B	−0.0871	0.6374	0.3814	0.155*	
H2C	−0.2178	0.5675	0.3670	0.155*	
O3	0.1088 (3)	0.4024 (2)	0.05598 (16)	0.1007 (11)	
C3	−0.0342 (4)	0.5115 (3)	0.32994 (19)	0.0676 (11)	
H3A	0.0523	0.5415	0.3114	0.081*	
C4	−0.0271 (3)	0.4105 (2)	0.22794 (18)	0.0540 (9)	
C5	−0.1076 (3)	0.3415 (2)	0.18197 (18)	0.0519 (9)	
C6	−0.1956 (4)	0.2662 (3)	0.2058 (2)	0.0698 (11)	
H6A	−0.2102	0.2600	0.2516	0.084*	
C7	−0.2619 (4)	0.2004 (3)	0.1630 (2)	0.0793 (12)	
H7A	−0.3206	0.1500	0.1798	0.095*	
C8	−0.2420 (4)	0.2086 (3)	0.0961 (2)	0.0736 (11)	
H8A	−0.2884	0.1636	0.0679	0.088*	
C9	−0.1545 (4)	0.2819 (3)	0.0684 (2)	0.0642 (10)	
C10	−0.0889 (3)	0.3463 (2)	0.11363 (19)	0.0540 (9)	
C11	−0.1371 (5)	0.2899 (3)	−0.0058 (2)	0.0973 (15)	
H11A	−0.0743	0.3448	−0.0160	0.146*	
H11B	−0.2279	0.3020	−0.0260	0.146*	
H11C	−0.0981	0.2282	−0.0228	0.146*	

### Atomic displacement parameters (Å^2^)

**Table:**

	*U*^11^	*U*^22^	*U*^33^	*U*^12^	*U*^13^	*U*^23^
O1	0.0298 (12)	0.130 (2)	0.0802 (19)	−0.0125 (14)	0.0014 (13)	−0.0014 (17)
N1	0.0309 (14)	0.083 (2)	0.079 (2)	−0.0139 (15)	0.0058 (16)	−0.0135 (18)
C1	0.146 (5)	0.126 (4)	0.107 (4)	0.021 (4)	−0.047 (4)	0.009 (4)
O2	0.096 (2)	0.0519 (16)	0.119 (3)	−0.0036 (16)	0.0068 (18)	0.0099 (17)
N2	0.0583 (19)	0.064 (2)	0.068 (2)	−0.0068 (18)	−0.0004 (17)	−0.0036 (17)
C2	0.106 (4)	0.079 (3)	0.125 (4)	0.010 (3)	−0.028 (3)	−0.028 (3)
O3	0.078 (2)	0.114 (2)	0.111 (2)	−0.0197 (18)	0.0431 (19)	−0.0240 (19)
C3	0.053 (2)	0.084 (3)	0.066 (3)	−0.019 (2)	0.001 (2)	−0.005 (2)
C4	0.0306 (17)	0.064 (2)	0.068 (2)	−0.0026 (17)	−0.0016 (18)	0.009 (2)
C5	0.0355 (17)	0.049 (2)	0.071 (2)	−0.0031 (16)	0.0008 (17)	0.0032 (19)
C6	0.058 (2)	0.074 (3)	0.077 (3)	−0.011 (2)	0.002 (2)	0.005 (2)
C7	0.076 (3)	0.065 (3)	0.096 (3)	−0.025 (2)	−0.003 (3)	0.005 (3)
C8	0.070 (3)	0.060 (2)	0.091 (3)	−0.011 (2)	−0.014 (3)	−0.004 (2)
C9	0.061 (2)	0.054 (2)	0.078 (3)	0.0006 (19)	−0.003 (2)	0.000 (2)
C10	0.0430 (19)	0.046 (2)	0.073 (3)	0.0032 (17)	0.0025 (18)	0.0047 (19)
C11	0.115 (4)	0.097 (3)	0.080 (3)	−0.018 (3)	−0.006 (3)	−0.010 (3)

### Geometric parameters (Å, °)

**Table:**

O1—C4	1.235 (3)	C3—H3A	0.9800
N1—C4	1.318 (4)	C4—C5	1.503 (4)
N1—C3	1.457 (4)	C5—C6	1.382 (4)
N1—H1A	0.8600	C5—C10	1.383 (5)
C1—C3	1.488 (6)	C6—C7	1.375 (5)
C1—H1B	0.9600	C6—H6A	0.9300
C1—H1C	0.9600	C7—C8	1.357 (5)
C1—H1D	0.9600	C7—H7A	0.9300
O2—N2	1.203 (3)	C8—C9	1.391 (5)
N2—O3	1.218 (3)	C8—H8A	0.9300
N2—C10	1.475 (4)	C9—C10	1.391 (5)
C2—C3	1.491 (5)	C9—C11	1.499 (5)
C2—H2A	0.9600	C11—H11A	0.9600
C2—H2B	0.9600	C11—H11B	0.9600
C2—H2C	0.9600	C11—H11C	0.9600
			
C4—N1—C3	123.4 (3)	N1—C4—C5	116.6 (3)
C4—N1—H1A	118.3	C6—C5—C10	116.9 (3)
C3—N1—H1A	118.3	C6—C5—C4	122.0 (3)
C3—C1—H1B	109.5	C10—C5—C4	120.9 (3)
C3—C1—H1C	109.5	C7—C6—C5	120.9 (4)
H1B—C1—H1C	109.5	C7—C6—H6A	119.5
C3—C1—H1D	109.5	C5—C6—H6A	119.5
H1B—C1—H1D	109.5	C8—C7—C6	120.2 (4)
H1C—C1—H1D	109.5	C8—C7—H7A	119.9
O2—N2—O3	124.3 (3)	C6—C7—H7A	119.9
O2—N2—C10	117.5 (3)	C7—C8—C9	122.2 (4)
O3—N2—C10	118.2 (3)	C7—C8—H8A	118.9
C3—C2—H2A	109.5	C9—C8—H8A	118.9
C3—C2—H2B	109.5	C10—C9—C8	115.6 (4)
H2A—C2—H2B	109.5	C10—C9—C11	123.7 (4)
C3—C2—H2C	109.5	C8—C9—C11	120.7 (4)
H2A—C2—H2C	109.5	C5—C10—C9	124.1 (3)
H2B—C2—H2C	109.5	C5—C10—N2	118.0 (3)
N1—C3—C1	111.4 (3)	C9—C10—N2	117.8 (3)
N1—C3—C2	110.1 (3)	C9—C11—H11A	109.5
C1—C3—C2	111.3 (4)	C9—C11—H11B	109.5
N1—C3—H3A	108.0	H11A—C11—H11B	109.5
C1—C3—H3A	108.0	C9—C11—H11C	109.5
C2—C3—H3A	108.0	H11A—C11—H11C	109.5
O1—C4—N1	124.1 (3)	H11B—C11—H11C	109.5
O1—C4—C5	119.3 (3)		
			
C4—N1—C3—C1	−95.0 (4)	C7—C8—C9—C11	−178.9 (4)
C4—N1—C3—C2	141.1 (4)	C6—C5—C10—C9	1.1 (5)
C3—N1—C4—O1	−3.0 (6)	C4—C5—C10—C9	176.8 (3)
C3—N1—C4—C5	175.6 (3)	C6—C5—C10—N2	179.1 (3)
O1—C4—C5—C6	132.9 (4)	C4—C5—C10—N2	−5.2 (5)
N1—C4—C5—C6	−45.8 (4)	C8—C9—C10—C5	−0.8 (5)
O1—C4—C5—C10	−42.6 (5)	C11—C9—C10—C5	178.0 (4)
N1—C4—C5—C10	138.7 (3)	C8—C9—C10—N2	−178.8 (3)
C10—C5—C6—C7	−0.6 (5)	C11—C9—C10—N2	0.0 (5)
C4—C5—C6—C7	−176.3 (3)	O2—N2—C10—C5	−62.5 (4)
C5—C6—C7—C8	−0.2 (6)	O3—N2—C10—C5	118.6 (4)
C6—C7—C8—C9	0.5 (6)	O2—N2—C10—C9	115.6 (4)
C7—C8—C9—C10	0.0 (6)	O3—N2—C10—C9	−63.3 (4)

### Hydrogen-bond geometry (Å, °)

**Table:**

*D*—H···*A*	*D*—H	H···*A*	*D*···*A*	*D*—H···*A*
N1—H1A···O1^i^	0.86	2.00	2.855 (3)	173
C11—H11A···O3	0.96	2.37	3.021 (5)	124

Symmetry codes: (i) *x*−1/2, *y*, −*z*+1/2.
